# Gemeinsame Perspektiven Humanistischer Psychotherapien in Covid-Zeiten

**DOI:** 10.1007/s00729-022-00214-8

**Published:** 2022-12-13

**Authors:** Ursula Grillmeier-Rehder, Karoline Hochreiter, Ida-Maria Kisler, Christian Korunka, Brigitte Schigl

**Affiliations:** 1 Integrative Gestaltherapie (IG) Wien, Wien, Österreich; 2 Psychodrama, Universität Salzburg, Salzburg, Österreich; 3 Existenzanalyse und Logotherapie, Abile, Donauuniversität Krems, Krems, Österreich; 4 Personzentierte Psychotherapie (APG*IPS), Universität Wien, Wien, Österreich; 5 Integrative Psychotherapie, Donauuniversität Krems, Krems, Österreich

**Keywords:** Covid-19, Humanistische Psychotherapien, Menschenbild, Therapeutische Schulen, Covid-19, Humanistic psychotherapies, Conception of man, Therapeutic schools.

## Abstract

Der Beitrag ist ein Versuch, das Spannungsfeld von Unterschieden und Gemeinsamkeiten der fünf großen humanistischen Schulen (Gestalt, Psychodrama, Personzentrierte PT, Logotherapie und Existenzanalyse, Integrative Therapie) in Bezug auf ein relevantes gesellschaftliches Themenfeld auszuloten. Die Covid-19 Pandemie stellt für die gesamte Psychotherapie eine große Herausforderung dar. Viele Menschen leiden unter den zahlreichen Auswirkungen der Pandemie. Aber auch die therapeutische Praxis hat sich dadurch verändert. Wir suchen in diesem Beitrag nach Antworten aus der Sicht der humanistischen Psychotherapieverfahren.

Im ersten Teil werden schulenspezifische Zugänge in Bezug auf die Auswirkungen der Pandemie vorgestellt. Hier unterscheiden sich die Schulen in Theorie, Sprache und Schwerpunktsetzungen. Anschließend versuchen wir die Gemeinsamkeiten unserer Ansätze zu erarbeiten. Im zweiten Teil werden Gemeinsamkeiten humanistischer Ansätze in Bezug auf den Umgang mit der Pandemie erarbeitet. Dazu gehören Aspekte des Menschenbildes, philosophische Verankerungen, die Spannungsfelder von Freiheit/Verantwortung und Autonomie/Bezogenheit und die Rolle der therapeutischen Beziehung.

## Abstract (en)

The article is an attempt to explore the dialectics between differences and commonalities of the five major humanistic schools (Gestalt, Psychodrama, Person-Centered PT, Logotherapy and Existential Analysis, Integrative Therapy) in relation to relevant social issues. The Covid-19 pandemic presents a significant challenge for psychotherapy. Many people are suffering from the numerous effects of the pandemic. But it has also changed therapeutic practice. In this paper, we seek answers from the perspective of humanistic psychotherapy practices.

In the first part, school-specific approaches are presented in relation to the impact of the pandemic. Here, the schools differ in theory, language, and therapeutic emphasis. In the second part, commonalities of humanistic approaches in relation to dealing with the pandemic are elaborated. These include aspects of the concept of humanity, philosophical anchors, dialectics of freedom/responsibility and autonomy/relationality, and the role of the therapeutic relationship.

## Die Ausgangslage

Ein Autor*innenkollektiv aus den fünf Schulen humanistischer Psychotherapien (Gestalt, Psychodrama, Personzentrierte PT, Logotherapie und Existenzanalyse, Integrative Therapie) versucht in diesem Beitrag, Antworten auf die Herausforderungen der Pandemie aus der Sicht ihrer eigenen Schulen und darauf aufbauend gemeinsame humanistische Perspektiven zu entwickeln. Ein solches Vorhaben ist ein erstmaliger, innovativer Schritt in der Geschichte der humanistischen Verfahren in Österreich – die Arbeitsgruppe wurde ins Leben gerufen, um gemeinsame Perspektiven für eine Identität eines humanistischen Clusters zu entwickeln.

Der vorliegende Beitrag, der aus diesen Diskussionen entstanden ist, stellt hier einen ersten Versuch dar. Wir haben dafür die Vorgangsweise gewählt, den Versuch der Entwicklung gemeinsamer Perspektiven an einem aktuellen und für die Psychotherapie relevanten Thema zu bearbeiten. Es war daher naheliegend, sich mit der Covid-19 Pandemie zu beschäftigen, die ja für jede/n einzelne/n und für die Gesellschaft mit zahlreichen Bedrohungen und Herausforderungen verbunden ist. Psychotherapie kann hier einen wichtigen Beitrag leisten, indem einerseits Erklärungsansätze zum Verständnis problematischer individueller, aber auch gesellschaftlicher Entwicklungen erarbeitet und andererseits Antworten auf diese Herausforderungen gefunden werden können.

Die Pandemie hat neben den direkten gesundheitlichen Konsequenzen der Erkrankung zahlreiche darüber hinaus gehende negative Auswirkungen. Viele Menschen leiden unter den ökonomischen Folgen der Pandemie. Internationale Studien verweisen auf Zunahmen von Ängsten, Schlafstörungen und weiteren psychischen Auswirkungen der Krise in weiten Teilen der Bevölkerung (z. B. Li et al. 2021; Teufel et al. 2022). Nach zahlreichen empirischen Befunden werden insbesondere bei Kindern und Jugendlichen teilweise ausgeprägte Zunahmen von klinischen Störungsbildern verzeichnet (z. B. Ravens-Sieverer et al. 2021).

In der Pandemie wurden für viele Menschen Sicherheiten und bisher vertraute Lebensgewohnheiten mit einem Schlag außer Kraft gesetzt. Gewissheiten, die bis dahin für selbstverständlich gehalten wurden, wie der Atemluft vertrauen oder einen Freund mit einer Umarmung begrüßen zu können, waren plötzlich zerstört (Spagnuolo-Lobb 2021).

In der therapeutischen Praxis finden diese Ängste ihren Platz. Wir können beobachten, dass Patient*innen gedanklich mit Pro- und Kontraargumenten hinsichtlich gesundheitspolitischer Maßnahmen (Impfung, Lockdown, Masken …) beschäftigt sind. Wir begegnen Patient*innen aus einander entfremdeten Lagern, die sich von den jeweils anderen gemobbt und bedroht fühlen, deren Freundschaften zerbrechen, in deren Familien Gräben aufreißen. Welche Hilfestellung haben wir im Rahmen der Psychotherapie hier zur Verfügung? Und wie lässt sich in der psychotherapeutischen Arbeit ein dialogischer Ansatz verwirklichen, wenn die Positionen verhärtet sind – insbesondere da die eigene unmittelbare existenzielle Betroffenheit der Therapeut*in ebenfalls Teil der Situation ist. Psychotherapeut*innen sind gleichermaßen bedroht von Corona, von den einengenden Maßnahmen, dem Verlust einer gesicherten, gesunden Atemluft, selbst in der therapeutischen Situation – und inzwischen auch vom immer größeren Bedrohungsgefühl durch den Krieg in Europa.

Die humanistischen Psychotherapieverfahren weisen sowohl in ihrer Praxeologie als auch in ihren theoretischen Fundierungen klare Unterschiede auf, durch die sie sich nicht selbstverständlich in einem einzigen übergreifenden Ansatz verorten lassen. Im ersten Teil unseres Beitrages beschreiben wir kurz Zugänge zu diesen Themenstellungen aus der Sicht der fünf humanistischen Psychotherapieverfahren und zeigen jeweils spezifische Zugänge. Im zweiten Teil versuchen wir gemeinsame Antworten auf die Herausforderungen der Pandemie aus einer schulen-übergreifenden humanistischen Perspektive zu entwickeln. Diese gemeinsamen Antworten wurden in Diskussionen innerhalb der Arbeitsgruppe entwickelt.

## Die Krise aus Sicht der einzelnen Humanistischen Verfahren

### Gestalttherapie

Gestalttherapeutische Grundkonzepte wie das Kontakt-Prozess-Modell, das Modell der Gestaltbildung vor einem Hintergrund sowie der Selbstorganisation des Organismus im Umweltfeld und die existenziell-dialogische Sicht auf den Menschen, können zum Verständnis der Phänomene während der Pandemie beitragen (Votsmeier-Röhr und Wulf 2017).

Bezugnehmend auf den eingangs geschilderten Verlust des vertrauten, sicheren Erfahrungshintergrundes, ändert sich auch die Art der Kontaktaufnahme mit der Umwelt.

Wenn die Umgebung als feindlich und ungreifbar wahrgenommen wird, erschöpft sich die Auseinandersetzung mit dem Problem in der beständigen mentalen Verarbeitung neuer, medial zugeführter Information. Der lebendige Input, den wir aus unseren sensorischen Kontaktkanälen beziehen, geht verloren. Angst blockiert den Kontakt und limitiert die Bedeutungskreation (Cavaleri 2020).

Das Bedürfnis, eine klare, eindeutige Figur zu erschaffen, führt dazu, dass durch Weglassen und phantasievolles Ergänzen dessen, was nicht vollständig wahrgenommen werden kann, einfachere, verständlichere Figuren entstehen, mit der die Welt wieder kontrollierbar erscheint (Heyer 2021).

In der psychotherapeutischen Praxis führt die dialogische Haltung zur Aktivierung eines authentischen Interesses an der anderen Person, trotz aller Unterschiede. So kann die Kontakterfahrung wieder mit der intersubjektiv erlebten, gegenwärtigen Wirklichkeit verbunden und adaptive Kräfte aktiviert werden.

Die gemeinsame Untersuchung des individuellen Hintergrundes oder Kontextes ermöglicht es den Kommunikationspartner*innen, sich unabhängig von der als „Lösung“ gefundenen Meinung als ganzer Mensch gesehen zu fühlen. Dies kann dazu beitragen, die eigene Position differenzierter zu betrachten, aber auch die Gründe für die anderen Positionen zulassen zu können.

Der ganzheitliche Ansatz der Gestalttherapie ermöglicht es, auch die physiologische Erfahrungsebene ins Kontakt-Gewahrsein einzubeziehen und sich auf diese Weise in der subjektiven Realität zu verankern.

Jede Kontaktnahme ist intentional auf etwas gerichtet. Die Patient*in in ihrer Aktivität wahrzunehmen, z. B. wie sie ihre Situation gestaltet, wie sie sich auf ihr Gegenüber bezieht, so dass sie sich als Agens der eigenen Lösung fühlen kann, reaktiviert die Fähigkeit, selbstwirksam mit der Umgebung zu interagieren und sich zumindest für den Augenblick und im Rahmen der gegebenen Einschränkungen ganz und spontan erleben zu können (Spagnuolo-Lobb 2021; Bloom 2020).

### Integrative Therapie

Die Integrative Therapie (IT) versteht sich als ein schulenübergreifendes Psychotherapie-Verfahren. Es verbindet v. a. tiefenpsychologische, leib- und bewegungstherapeutische Ansätze mit humanistischen Wurzeln v. a. aus der Gestalttherapie und dem Psychodrama sowie philosophischen und neurowissenschaftlichen Erkenntnissen. Das IT-Menschen- und Weltbild besagt, dass wir in einer gemeinsamen Lebenswelt, dem sozialen und ökologischen Kontext in der jeweiligen historischen Zeit (Kontinuum) leben. Diese Lebenswelt hat sich in den Zeiten der Pandemie für viele radikal geändert, große Anpassungsleistungen werden sowohl von Patient*innen wie ihren Therapeut*innen abverlangt.

Alle fünf Säulen unserer Identität (Petzold 2003) sind betroffen: Die Leiblichkeit war unmittelbar durch die unbekannte Erkrankung bedroht, der physische Bewegungsradius eingeschränkt. Besonders stark waren die Veränderungen im Bereich der sozialen Netzwerke – hier wurden wir von Freund*innen und Familienmitgliedern abgeschnitten bzw. unbeschwerte Treffen verunmöglicht. Arbeit und Freizeit waren starken Veränderungen unterworfen: Manche hatten sehr viel mehr, andere durch Betriebsschließungen keine Arbeit und gewohnte Freizeitaktivitäten waren verunmöglicht. Die materielle Sicherheit war für viele beeinträchtigt oder zumindest die Sorge davor Thema. In Bezug auf Sinn und Werte wurden viele sehr deutlich mit existentiellen Themen von Krankheit und Tod konfrontiert. Auch unser Verhältnis zum Staat wurde neu diskutiert, hatten doch die meisten Menschen Freiheitsbeschränkungen und so viele wechselnde Gebote noch nie in ihrem Leben erfahren. In einer Diagnose, welche Identitätsbereiche individuell besonders belastet sind, lassen sich psychotherapeutisch erste Schwerpunkte setzen.

Integrative Therapie arbeitet mit Patient*innen im verbalen und nonverbalen Austausch unter Nutzung kreativer Methoden. Wichtigstes agens movens der Therapie ist die therapeutische Beziehung, die sich in Prozessen „inter-subjektiver Ko-respondenz“ (Petzold 2017) entwickelt. Wir gehen von der aktuellen Lebenssituation mit ihren Belastungen aus und arbeiten mit dezidierter Zukunftsorientierung (Stefan 2020) in vier Wegen der Heilung und Förderung: Für die Belastungen der Pandemie kann der erste Weg einer Bewusstseinsarbeit v. a. für die Konstituierung von Sinnzusammenhängen genutzt werden – „worauf macht mich die Problematik aufmerksam, was kann ich daraus lernen?“. In der erlebens- und übungszentrierten Modalität können Alternativen zu bis jetzt geübten Verhaltensweisen exploriert, ausprobiert und idealiter in den Alltag übernommen werden. Die vierte Dimension der Heilung und Förderung schließlich knüpft an die gemeinsame Existenz in der Lebenswelt an und kann durch die von Therapeut*in und Patient*in geteilte Erfahrung der Schwierigkeiten Verständnis und Solidaritätserfahrung beinhalten. Die Begegnung zweier oder in der Gruppentherapie mehrerer Subjekte, die die schwierige Situation zu bewältigen haben, führt zur kognitiven und emotionalen Erfahrung, mit den Herausforderungen der Situation nicht alleine zu sein. So kann im Idealfall nicht nur ein „Coping“ mit der problematischen Situation, sondern „Creating“ – der kreative Umgang damit, der Neues schafft und Perspektiven öffnet, gefunden werden.

### Existenzanalyse und Logotherapie nach Viktor E. Frankl

Existenzanalyse und Logotherapie trägt mit ihrem Sinn orientierten Ansatz zu einem konstruktiven und an der Gesundheit orientierten Menschenbild bei, indem sie auf die spezifisch humane noetische (geistige) Dimension (Frankl 1994) Bezug nimmt. In ihr eröffnet sich uns die Freiheit, zu physischen und psychischen Gegebenheiten Stellung zu beziehen, uns abzugrenzen und zu opponieren. Die „Trotzmacht des Geistes“ (Frankl 1998) ermöglicht die therapeutische Arbeit an Einstellungen im Hinblick auf Situationen, die wir direkt nicht beeinflussen können. Die Logotherapie stellt dafür Methoden zur Selbstdistanzierung und Selbsttranszendenz zur Verfügung, wie etwa die Dereflexion (Biller und de Lourdes Stiegeler 2008), die Einstellungsmodulation und die Bezugnahme auf Humor (ebda).

Moderne Forschung bestätigt V. Frankls Annahmen: In einer empirischen Studie konnte gezeigt werden, dass Menschen, die ein sinnorientiertes Leben führen und die Fähigkeit zur Selbstkontrolle besitzen, den mit der Pandemie verbundenen Stress besser bewältigen können (Schnell und Krampe 2020).

In der existenzanalytischen Arbeit wird ein Raum angeboten, in dem Patient*innen Zeit bekommen, sich selbst und das eigene Geworden-Sein in einer vertrauensvollen therapeutischen Beziehung, in der sie als Menschen in ihrer Einmaligkeit und Einzigartigkeit (an)erkannt werden, zu reflektieren. Leid existiert und verweist gleichzeitig darauf, dass die Welt eigentlich anders sein soll. Indem diese Sein-Sollen-Spannung thematisiert wird, wird auch der Weg für eine rationale Distanzierung vorbereitet (Frankl 1998).

In der logotherapeutischen Arbeit erfolgt eine Hinwendung zu den umfassenden existenziellen Fragen „Wer bin ich?“ (Selbstbild) und „Welcher Mensch will ich sein?“ („achtsame Selbstwahrnehmung“). Letztere Frage bildet die Grundlage für eine existenzielle Entscheidung. Bezogen auf z. B. unterschiedliche Anschauungen zum Thema Covid-Impfung liegt der Fokus unserer Patient*innen dann nicht mehr auf der Frage: „Soll man sich impfen lassen?“, vielmehr „Wie wird mein Leben aussehen, wenn ich mich impfen lasse? Wie würde das mein Leben verändern? Und wie sehr will ich dieser Mensch sein?“. Im logotherapeutisch angeleiteten Prozess der Stellungnahme zu sich selbst, können Patient*innen, sich selbst transzendierend, ihre Freiheit erfahren und Möglichkeiten ausloten, verantwortlich mit dieser umzugehen.

Bewegt man sich mit Patient*innen in jenen Bereich von Wertvorstellungen, welche auf Zukünftiges, erst zu Verwirklichendes verweisen, verlässt man zum Teil den Bereich empirischer Realität und betritt den Raum existenzieller Möglichkeiten. Je besser diese ausgelotet werden, umso besser kann der individuelle und einmalige Prozess menschlicher Reifung stattfinden.

### Personzentrierte Psychotherapie

Die Personzentrierte Psychotherapie bietet mit ihrem zentralen Konzept der notwendigen und hinreichenden Bedingungen von Psychotherapie (Rogers 1957) einen Ausgangspunkt für die Auseinandersetzung mit Covid-Themen. Im spezifischen Erfahrungskontext der Pandemie sind allerdings einige Besonderheiten zu beachten, die sich auf die therapeutische Beziehung auswirken können. (1) Wir befinden uns im gesamten Verlauf der Pandemie in einem in dieser Form noch nie dagewesenen gemeinsamen Erfahrungskontext. (2) Spätestens im Verlauf der Pandemie haben sich Einstellungen und Haltungen herausgebildet, die oft zu sehr deutlichen Positionierungen geführt haben. Was bedeutet dies nun für die drei zentralen Aspekte der therapeutischen Beziehung?

Akzeptanz (Unconditional Positive Regard) beschreibt eine grundsätzliche Haltung der Akzeptanz einer Person gegenüber, mit all ihren Einstellungen, Haltungen und Erlebenszuständen ohne jegliche Wertung seitens der Therapeut*in (Rogers 1951). Das Erleben des Angenommenseins ohne Vorbedingungen ist für Patient*innen eine intensive und heilende Erfahrung. Es liegt auf der Hand, dass dieser Haltung in der aktuellen Situation der polarisierten Einstellungen eine ganz besondere Bedeutung zukommt (Molyneux 2021a), wenn beispielsweise ein/e Impfgegner*in ihren Ansichten ohne Wertung angenommen wird.

Empathie beschreibt das einfühlsame Verstehen der inneren Welt des Gegenübers, mit all seinen Gefühlen, Gedanken und Einstellungen, das Kommunizieren dieses umfassenden Verstehens, das Erleben der Welt der Klient*in, als ob es seine eigene wäre (Rogers 1957). Im Zusammenhang mit den Themen der Pandemie bedeutet dies ein tiefes Verstehen der Welt des anderen, sei es auch die Welt eines radikalen Impfgegners mit all seinen existenziellen Sorgen und Ängsten. Empathisches Verstehen liefert die Basis für personale Begegnung, sie liefert einen, vielleicht den wesentlichen Beitrag zum Verstanden werden, zum Brückenschlag in einer polarisierten Welt (Molyneux 2021b).

Kongruenz bedeutet die Authentizität und Echtheit des Verstehenden, in Bezug auf sein eigenes Erleben des Gegenübers und seine eigenen Gefühle und Haltungen (Rogers 1957). Eine kongruente Haltung ist die Grundlage dafür, dass Akzeptanz und Verstehen im Sinne einer personalen Begegnung zur Entfaltung kommen können. Sie ist aber auch eine besondere Herausforderung und ein hoher Anspruch für Psychotherapeut*innen, gerade in einer Situation gemeinsamer Betroffenheit, in der auch sehr unterschiedliche Einstellungen sichtbar werden. Um personale Begegnung in der Psychotherapie erfolgreich umzusetzen, sind auch eigene erstarrte Selbstkonzeptanteile (die gerade in andauernden Krisensituation entstehen können) zu hinterfragen.

Wenn es aber gelingt, die drei notwendigen und hinreichenden Bedingungen der therapeutischen Beziehung gemeinsam umzusetzen, dann wird Begegnung und Veränderung gerade auch in der derzeitigen Situation möglich (Molyneux 2021c). Es besteht die Chance und Wahrscheinlichkeit aus den eigenen „Echokammern“ in der pandemischen Entwicklung auszusteigen, und sich auch in der aktuellen Situation vom Rätsel des Gegenübers (im Sinne von Levinas) berühren zu lassen.

### Psychodrama

Psychodramatiker*innen richten ihre Aufmerksamkeit sowohl auf die angemessene Gestaltung der therapeutischen Beziehung (Schacht 2018) als auch auf das Erkennen und Neugestalten der komplexen Szene (Schacht 2009), in der sich die Patient*innen erleben. Für das Gelingen der therapeutischen Beziehung gelten wie in den anderen humanistischen Therapieformen die Grundprinzipien von Akzeptanz, Empathie und Kongruenz. Für die thematische Arbeit bedienen sich Psychodramatiker*innen variabler Mittel: Szenische Arbeit im Raum, auf dem Tisch oder im Gespräch kann unter Einbeziehung anderer Menschen (in der Psychodrama-Gruppe) als Mitspieler*innen, oder unter Verwendung von Intermediärobjekten (Gegenstände, Schreiben, Zeichnen, etc.) erfolgen (Stadler und Kern 2010). Wesentlich wirksam ist dabei der (verkörperte) Perspektivenwechsel (Rollenwechsel).

Am Beginn der szenischen Arbeit im Einzel- oder Gruppensetting steht der Szenenaufbau: Wo findet die Szene statt? Wer oder was ist daran beteiligt? Handelt es sich um Realität, Phantasie, Traum? Was wird abgehandelt? Die Corona-Pandemie brachte Anforderungen mit sich, die für Patient*innen graduell unterschiedlich schwierig zu bewältigen waren. Tendenziell wurde beispielsweise von Patient*innen stärker ihre Position gegenüber den Corona-Maßnahmen thematisiert als ihre Positionierung zum Corona-Virus selbst. Manchen fiel es leichter, pauschal empört zu sein auf „die Politiker“, als sich mit dem Virus selbst zu konfrontieren. In diesem Fall können „die Politiker“ auf die Tisch- oder Raumbühne geholt werden. Nach einer ersten, durchaus unzensuriert möglichen Beschwerdeführung oder Anklage wird ein Rollenwechsel mit „den Politikern“ angeleitet, die Klient*in verkörpert diese Rolle dabei selbst, antwortet auf die vorgebrachte Beschwerde. Mit mehreren solchen Rollenwechseln wird eine zunehmend konstruktive Auseinandersetzung möglich. Zu einem geeigneten Zeitpunkt wird auch das Corona-Virus selbst in die Szene eingebracht und eine direkte Auseinandersetzung mit diesem ebenfalls in mehreren Rollenwechseln und anderen Bühnentechniken angeleitet, wobei Angst vor Tod und Krankheit, Wut, Aggression, Verzweiflung etc. zum Ausdruck kommen und bearbeitet werden können. Das Psychodrama bietet auch die Möglichkeit, die Pandemie-Thematik überindividuell als allgemein-gesellschaftlich zu bearbeiten. Dafür gibt es das Arrangement Soziodrama (Buckel et al. 2021), in dem unterschiedliche Personengruppen repräsentiert werden und miteinander in Interaktion treten. Leider hat Corona die Möglichkeit für Gruppenarbeit wesentlich eingeschränkt. Jeder Bühnenarbeit folgt jedenfalls eine Integrationsphase mit Sharing, Identifikationsfeedback und Zuschauerfeedback. Diese fördern zusätzlich die Verbundenheit zwischen den Anwesenden. Wurde in der Pandemie ein Trauma getriggert, gelten bei der Bearbeitung die traumaspezifischen Modifikationen (Pruckner 2018).

## Gemeinsame Perspektiven aus der Sicht humanistischer Psychotherapien

Die obigen Skizzen aus der Sicht der fünf Verfahren zeigen unterschiedliche verfahrensspezifische Zugänge im Umgang mit den psychischen Auswirkungen der aktuellen Krise. Es finden sich aber durchaus einige wesentliche Gemeinsamkeiten. Wir können auf der Grundlage unserer Diskussionen hier sechs Grundpositionen des humanistischen Ansatzes als Ausgangspunkte identifizieren:

1. Die humanistischen Psychotherapieverfahren besitzen eine philosophische Grundlage, aus der sich ein *Menschen- und Weltbild mit einem impliziten Wertekanon* ableitet. Diesem immanent ist der Blick auf die Einbettung des einzelnen Menschen in sein soziales und ökologisches Umfeld.
  Daraus folgt neben der Bezugnahme auf die individuelle Ebene auch eine Stellungnahme zu gesellschafts-politischen Entwicklungen wie etwa eine kritische Position gegenüber den negativen Auswirkungen des globalen Kapitalismus.
2. Menschliches Leben und Überleben vollzieht sich seit jeher in Gemeinschaften, in sozialen Mikroökologien, in denen Menschen füreinander Umwelt und Bezug sind. Der *existenzphilosophische* Ansatz besagt, dass wir als Lebewesen eine *gemeinsame* Welt teilen, in die wir geworfen sind. Diese Welt können wir nur gemeinsam gestalten. Als soziale Wesen brauchen wir die Anderen. Dies stellt eine besondere Herausforderung an Psychotherapie in Covid-Zeiten dar: Wir sind gleichermaßen mit dem kollektiven Phänomen der Virusbedrohung konfrontiert und teilen die zeitgeschichtliche Erfahrung und Erschütterung, die unser aller Leben unwiderruflich und existenziell veränderte. Die Betroffenheit mag unterschiedlich sein, doch erleben wir alle eine Vertreibung aus der gewohnten Art zwischenmenschlicher Bezüge der Vor-Corona-Zeit und müssen uns selbst neu orientieren.
  Ein Sich-Abgrenzen, wie oft in Gesundheitsberufen gefordert, ist vielfach nicht mehr möglich. Es wird klar, dass wir angrenzend arbeiten müssen, weil wir als Individuen gemeinsam mit den Herausforderungen unserer Zeitläufe umgehen müssen. Erschreckend klar wird, wie umfassend diese geteilte Wirklichkeit ist, die Psychotherapeut*innen und Patient*innen verbindet. Die Atemluft, die spontane Nähe zum Anderen, der öffentliche Raum, der geteilte Raum, sind mit einem Mal zur Gefahrenquelle geworden. Wir werden konfrontiert mit unserer existenziellen Verletzlichkeit als Mitgeschöpfe der Natur.
  Psychotherapeut*innen hatten die Herausforderung, dass sie mit denselben Fragen, Sorgen und Einschränkungen konfrontiert waren, wie ihre Patient*innen, doch gleichzeitig Information, Zuversicht und Ruhe vermitteln sollten. Im therapeutischen Kontext müssen die Regeln neu gesetzt werden, neue Settings wie Online- und Telefontherapie entwickelt werden, die unmittelbare Präsenz ist von Masken- und Abstandspflicht und anderen Schutzmaßnahmen in Therapie beeinflusst. Therapie-methodische Möglichkeiten müssen an diese Einschränkungen angepasst werden.
3. Die Fähigkeit, in *Freiheit* Entscheidungen treffen zu können und *Verantwortung* für diese zu übernehmen, ist in einem humanistischen Menschenbild im menschlichen Wesen verankert. Dies drückt sich in der Wahrnehmung seiner Einzigartigkeit und Fähigkeit zur Sinnfindung aus, in der Fähigkeit zu Resonanz (Rosa 2019) und zu sozialer Konstitution einer geteilten, gemeinsamen Wirklichkeit, sowie der Fähigkeit, sich im Anderen wesenhaft wiederzuerkennen. Wir sind als Beziehungswesen in der Lage, einander zu begegnen und aus dieser Begegnung neue Lebendigkeit und Sinn zu generieren. Dies impliziert Selbstverantwortlichkeit und das Bewusstsein für soziale und ökologische Verantwortung, aus der in der globalen Krise niemand aussteigen kann.
  Wir stehen vor der Paradoxie, dass Freiheit auch darin besteht, unser gegenseitiges Einander-Brauchen anzuerkennen und Wege zu finden, einen immer wieder herzustellenden Ausgleich zu schaffen, um den gemeinsamen Boden zu stärken und zu schützen – den Boden unserer Beziehungen zueinander und den Boden unserer Beziehung zu der uns tragenden natürlichen Umwelt.
  Die persönliche, soziale und ökologische Risikoabwägung z. B. in Bezug auf die Frage des Impfens und die Einhaltung von Schutzmaßnahmen werden Themen in der Psychotherapie, die Frage der sozialen Verantwortung kann so in den Blick genommen und unterstützt werden. Welche Gewichtung gibt die/der Einzelne dieser Frage? Mit wem mache ich gemeinsame Sache? Welche Ziele verfolge ich langfristig? Welche Folgen hat mein Handeln? Wie gut sind Selbstwert und Selbstwirksamkeit entwickelt, um mit den kollektiven Ängsten umzugehen und einen solidarischen Blick behalten bzw. entwickeln zu können?
4. Zentral für den humanistischen Ansatz ist die Bedeutung einer nicht-utilitaristischen, *dialogischen, wechselseitigen Beziehung von Mensch zu Mensch*. Daraus folgt für die Praxis – innerhalb bestimmter Rollen – eine Grundhaltung bedingungsloser Wertschätzung und Respekts für das So-Sein des Anderen und der Blick auf die *therapeutische Beziehung* als essentielles *heilendes Agens Movens*, die über eine „Arbeits“beziehung (Alliance) hinausgeht: Der Mensch entwickelt sich an und mit anderen menschlichen Wesen durch Momente der *intersubjektiven, personalen Begegnung und Korrespondenz*. Übertragungen werden bearbeitet, um authentische Begegnung zu ermöglichen.
  Durch die notwendigen Anpassungen an die Corona-Schutzmaßnahmen wird nun diese therapeutische Haltung herausgefordert. Psychotherapeut*innen müssen sich auf die neuen Regelungen beziehen und diese in ihren therapeutischen Prozessen etablieren. Durch diese notwendige Positionierung kann die intersubjektive Kommunikation schwieriger werden. Die Position des*r Anderen als wertvoll zu erachten und auch völlig entgegengesetzten Haltungen wertschätzend und verständnisvoll zu begegnen, wird durch die unmittelbare Selbst-Betroffenheit erschwert. Die Corona-Maßnahmen verändern die Möglichkeiten, Nähe und Distanz spontan zu regulieren. Die existentielle Begegnung kann durch diese Prozesse beeinträchtigt werden. Allerdings – wenn sie dennoch gelingt, kann sie zu einer verstärkten Erfahrung der therapeutischen Begegnung über das Trennende hinweg werden.
5. Dem Menschen wird im humanistischen Denken eine innewohnende Tendenz zur *Aktualisierung seines Potentials* zugeschrieben, die bedeutet, dass er seine Entwicklungsbedürfnisse und -möglichkeiten nach seiner eigenen inneren Gesetzmäßigkeit in einem stetigen Prozess zu entfalten sucht. Das therapeutische Handeln fokussiert deshalb auf die Entfaltung von Potenzialen und *Zukunftsmöglichkeiten*.
  Diese Potenziale sind nicht normativ festgelegt und beinhalten die Erkenntnis- und Reflexionsfähigkeit, die Fähigkeit zu Phantasie, Kreativität und schöpferischer Gestaltung, sowie die Fähigkeit, andere Menschen empathisch mitfühlend wahrnehmen zu können und am Kontakt und Austausch mit der Umwelt zu wachsen. Insofern steht die Potentialorientierung nicht im Widerspruch zu einer Haltung, sich dem Leid, sowohl dem eigenen als auch dem der Anderen, zu stellen. Reflexionsfähigkeit und Nachdenklichkeit (im Sinne von Hanna Ahrendt) sind wesentliche gesellschaftliche Schutzfaktoren gegenüber demagogischer Verführung und der Vereinnahmung durch menschenverachtende politische Haltungen.
  Zukunftsorientierung ist in den Unsicherheiten und wechselnden Bedingungen der Covid-Krise besonders wichtig, um eine weiterreichende Perspektive über die aktuelle Bedrohungssituation hinaus zu entwickeln und neue Wirklichkeiten zu entwickeln. Die Fokussierung auf Potenziale gibt Hoffnung und stärkt in Krisenzeiten.
6. Der Lebensbogen des Menschen ist gekennzeichnet durch die *Dialektik* und das Spannungsfeld von *Autonomiebedürfnissen* und einem tiefen Grundbedürfnis nach *Bezogenheit und Verbundenheit*, zwischen deren Polen er ständig gefordert ist, eine Balance herzustellen.
  Die gesellschaftliche Entwicklung der letzten Jahrzehnte betont die Autonomie auf Kosten der Bezogenheit. So werden beispielsweise die Auswirkungen des Individualverkehrs marktwirtschaftlich nicht dem Verursacherprinzip zugeordnet, sondern deren soziale, ökologische und klimapolitischen Folgekosten auf die Gesellschaft ausgelagert (Blanck et al. 2020). Die gegenwärtigen Krisen fordern uns existenziell heraus, den Blick auf die Notwendigkeit sozialer Gerechtigkeit und gesellschaftliche Solidarität zu deren Bewältigung zu richten und dies könnte auch eine Chance sein, beide Elemente wieder mehr ins Gleichgewicht zu bringen.
  Die gesellschaftliche Entwicklung spiegelt sich auch auf der biografischen Ebene: Die aktuellen Haltungen von Personen in Krisen können in Bezug gesetzt werden zu ihrem Bindungserleben und der Geschichte ihrer Autonomieentwicklung: An dieser Stelle verunsicherte Menschen werden auch in der Covid-Krise leichter verängstigt sein oder sich in Abgrenzung gegen gemeinsame Regelungen stellen.

Die hier angestellten Überlegungen sind nicht nur für die Bewältigung der Covid-19 Pandemie gültig, sie haben Bedeutung in der Bewältigung verschiedener gesellschaftlichen Krisen, die wir leider derzeit erleben müssen. Humanistische Psychotherapien bieten mit ihrem emanzipativen und optimistischen Menschenbild und mit ihrer Fokussierung auf Potenziale und Möglichkeiten der Individuen konstruktive Antworten auf die Herausforderungen unserer Zeit. Unser Beitrag stellt dazu einen Annäherungsprozess im Spannungsfeld von Unterschieden und Gemeinsamkeiten innerhalb der humanistischen Verfahren dar.
